# Improved Aging Stability of Ethylene-Norbornene Composites Filled with Lawsone-Based Hybrid Pigment

**DOI:** 10.3390/polym11040723

**Published:** 2019-04-19

**Authors:** Anna Marzec, Bolesław Szadkowski

**Affiliations:** Institute of Polymer and Dye Technology, Faculty of Chemistry, Lodz University of Technology, Stefanowskiego 12/16, 90-924 Lodz, Poland; boleslaw.szadkowski@edu.p.lodz.pl

**Keywords:** ethylene-norbornene copolymer, lawsone, aluminum-magnesium hydroxycarbonate, solar irradiation, hybrid pigment

## Abstract

In this study, we produced a new organic-inorganic hybrid pigment based on a natural chromophore. Lawsone was selected as the active organic compound and incorporated into aluminum-magnesium hydroxycarbonate (LH). The hydroxynaphthoquinone derivative lawsone (*Lawsonia inermis L*.) is a naturally occurring dye, which is commonly used as a colorant because of its nontoxicity and biological functions. The structure and stability of the hybrid colorant were investigated using 27-Al solid-state nuclear magnetic resonance (NMR) spectroscopy, X-ray diffraction (XRD), secondary ion mass spectrometry (TOF-SIMS), thermogravimetric analysis (TGA), scanning electron microscopy (SEM) and UV-Vis spectroscopy. TOF-SIMS and ^27^Al NMR spectroscopy revealed interactions between the dye molecules and metal ions present in the LH host, confirming successful formation of an LH-based hybrid (LH/lawsone). In the next part of the study, we examined the effect of the hybrid pigment on the mechanical and thermal properties of ethylene-norbornene (EN) materials, as well as the aging resistance of the colored composites to irradiation across the full solar spectrum. Dynamic mechanical analysis (DMA) and the results of tensile break tests revealed that the EN+LH/lawsone composite had significantly better resistance to solar irradiation in comparison to EN and EN with an unmodified carrier.

## 1. Introduction

Many colorants are now recognized as carcinogenic or allergenic, leading to their disuse. There is growing interest therefore in replacing artificial colorants with natural compounds or identical synthetic dyes [1,2,3]. Naturally occurring substances as well as synthetic compounds are already widely used as colorants in various products, such as paints, plastics, fibers and rubbers. However, many dyes are unstable under polymer processing conditions (e.g., heat, pH changes, the presence of other additives), resulting in loss of color. Therefore, organic-inorganic hybrid materials have emerged as an alternative form novel colorant with desirable characteristics. For several decades, there has been sharp rise in the development of new organic-inorganic hybrid materials with unique or improved properties, such as enhanced resistance to migration and better chemical or thermal stability [4,5,6,7]. Recently, intercalated/adsorbed hybrid colorants have been shown to be outstanding multifunctional hybrid pigments, able to act as coloring agents and fillers in polymer materials. In comparison to non-colored composites, organic-inorganic hybrids containing polymers exhibit significant improvements in terms of several important properties, including mechanical strength, flammability, aging resistance and decreased gas permeability [8,9,10]. Zimmermann et al. [11] produced hybrid colorants by intercalation of zinc layered hydroxide salts (LHS) with anionic orange azo dyes, using a co-precipitation method. It was observed that HDPE filled with zinc LHS intercalated with azo dyes had improved mechanical properties, good interface bonding and better stability to UV irradiation in comparison to the reference samples.

Lawsone (*Lawsonia inermis L.*) is an active compound which can be obtained from the leaves of henna. According to industrial classifications, lawsone is Natural Orange 6 and CI 75480, chemically known as 2-hydroxy-1,4-naphthoquinone [12]. Henna has been used for centuries in many countries to dye hair and eyebrows and to paint the skin and fingernails [13,14]. Lawson, like other natural dyes, does not pose any hazard to health and is environmentally friendly, with slight chemical reactivity that does not have any adverse ecological effects. This natural colorant acts as a substantive dye for protein fiber as well as other textile fibers. It gives textile materials not only an orange color but also antibacterial properties [15,16]. Recently, lawsone has become more important due to its applications in medicine, as an astringent, antiseptic and antipyretic substance [17,18]. Yasin et al. [19] prepared a drug-inorganic nanostructured material using a pharmaceutically active compound of lawsone, which was intercalated between the layers of double hydroxide by co-precipitation and ion exchange methods. The results demonstrated that layered double hydroxide can be used as a carrier for lawsone with controlled delivery capability. This behavior was found to be dependent on the pH of the release medium.

In the present study, lawsone was selected as an active organic compound and stabilized onto aluminum-magnesium hydroxycarbonate (LH) by the precipitation method. In our previous research, we had used this method to obtain hybrid pigments based on anthraquinone and azo chromophores [20,21]. The host-dye interactions and morphology of the hybrid LH/lawsone pigment were characterized by TOF-SIMS, ^27^Al NMR spectroscopy, X-ray powder diffraction (XRD) and scanning electron microscopy (SEM). Next, ethylene-norbornene copolymer (EN) was filled with aluminum-magnesium hydroxycarbonate modified using the lawsone colorant. Control samples with unmodified aluminum-magnesium hydroxycarbonate filler and unfilled composite were also prepared for the purposes of comparison. The resulting EN composites were characterized using tensile tests, DMA and FT-IR spectroscopy before and after irradiation under the full sun spectrum.

## 2. Materials and Methods

### 2.1. Raw Materials

Lawsone (2-hydroxy-1,4-naphthoquinone), an orange dye, was provided by Sigma Aldrich (Schnelldorf, Germany). The dye was used to modify a PURAL^®^ MG 30 aluminum-magnesium hydroxycarbonate host (LH) (Al:Mg weight ratio 70:30), kindly supplied by Sasol company (Hamburg, Germany). The polymer matrix was ethylene-norbornene copolymer (EN) (with 40 wt% of bounded norbornene, density: 0.94 g/cm^3^, melt flow rate: 3 cm^3^/10 min, melting temperature of 84 °C and Vicat softening temperature of 64 °C), purchased from TOPAS Advanced Polymers (Frankfurt-Höchst, Germany).

### 2.2. Pigment Preparation

The modification of aluminum-magnesium hydroxycarbonate (LH) with lawsone was performed in an aquatic environment. First, a set amount of the lawsone (3 g) was dissolved in a solution of ethanol (20 mL) in deionized water (200 mL). Next, the mixture was subjected to ultrasonication for 30 min, before being heated to 75 °C. Subsequently, the aluminum-magnesium hydroxycarbonate host (17 g) was added and the solution was stirred continuously for 2.5 h at a constant temperature (75 °C). The precipitated hybrid pigment (LH/lawsone) slurry was filtered off, washed with deionized water and dried in an oven under a static air atmosphere.

### 2.3. Composite Preparation

**Figure 1:**
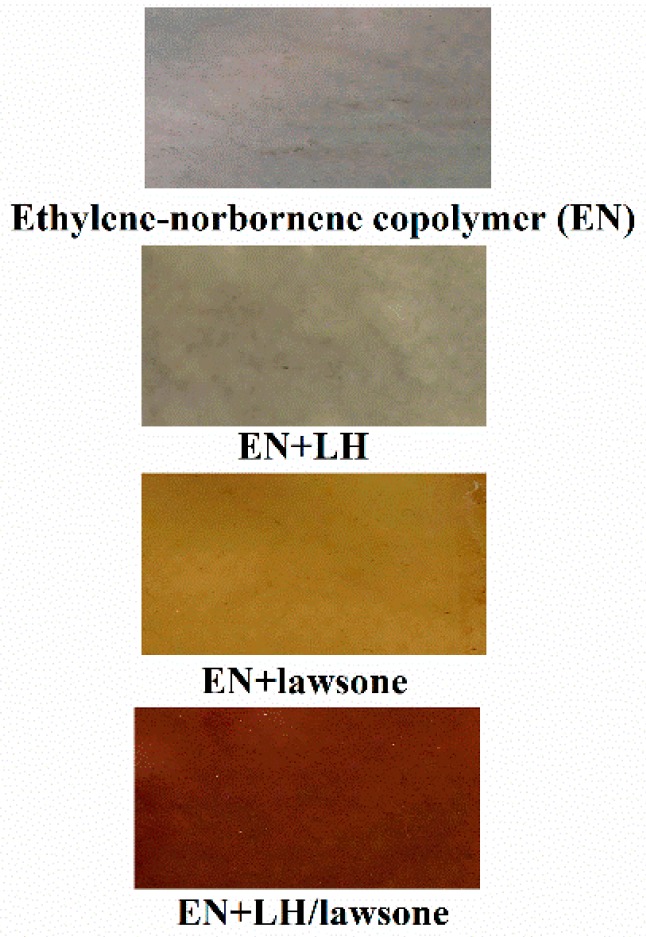
Digital photographs of the EN composites filled with LH, lawsone and LH/lawsone hybrid.

The polymer composites were prepared using a Brabender N50 laboratory measuring mixer. First, pure ethylene-norbornene copolymer (100 g) was processed using a rotor speed of 50 rpm at 120 °C. Next, the prepared hybrid pigment (3 g) was added. Mixing was continued for the next 15 min. The mixing curves for prepared compounds are shown in the Figure S1, Supplementary Material. After mixing, the compounds were pressed between two hot steel plates at the temperature of 120 ˚C for 15 min to obtain composite sheets with a thickness of about 1 mm. The obtained composite samples (Figure 1) were cooled freely on fresh air.

### 2.4. Pigment Characterization Techniques

Secondary ion mass spectrometry (TOF-SIMS) was used to study the interactions between the organic and inorganic components of the hybrid pigment. The TOF-SIMS spectra were recorded using a TOF-SIMS IV mass spectrometer (IONTOF GmbH, Muenster, Germany), equipped with a Bi liquid metal ion gun and a high mass resolution time of flight mass analyzer. The analyzed area of the sample was irradiated with pulses of 25 keV Bi^3+^ ions at a repetition rate of 10 kHz and with an average ion current of 0.4 pA. Solid state Nuclear Magnetic Resonance (MAS NMR) measurements were performed in a Bruker Avance III 400 WB (Rheinstetten, Germany) spectrometer operating at a resonance frequency of 104.26 MHz. X-ray diffraction analysis (XRD) was performed using an Analytical Pert Pro MPD diffractometer (Malvern Panalytical Ltd., Royston, UK). The XRD patterns were collected in the Bragg-Brentano reflecting geometry with (Cu Kα) radiation, in the range of 2*θ* = 2–70°. The thermal stability of the samples was assessed using a Thermogravimetric Analyzer (TA Instruments, Greifensee, Switzerland). The measurements were performed in the presence of argon in a temperature range of 25–600 °C, with a heating rate of 10 °C/min. The UV-Vis spectra were recorded using an Evolution 201/220 UV-Visible Spectrophotometer (Thermo Fisher Scientific, Waltham, MA, USA). The tests were conducted in a spectral window from 1100 to 200 nm at room temperature. The measurement specimens were in the form of solid-state powders. They were placed in a special powder cell holder. Before the measurements, the baseline was corrected using a special calibration adapter. The accuracy of the apparatus was ±0.8 nm and the repeatability was ≤0.05 nm. The morphology of the studied powder samples was inspected by scanning electron microscopy (SEM) using a LEO 1530 Gemini scanning electron microscope (Zeiss/LEO, Oberkochen, Germany).

### 2.5. Composite Characterization Techniques

Weather aging of the EN samples (in the form of composite sheets) was performed with light radiation of λ = 280–3000 nm using a Solar Climatic 340 instrument for 350 h. The weathering was based on two periods, simulating day and night conditions. The test timeline had the following parameters: day period—solar irradiation, temperature 70 °C, humidity 50%, duration 8 h; night period—temperature −20 °C, humidity 60%, duration 4 h.

The thermal decomposition (TGA) of the composites was investigated using a Thermogravimetric Analyzer (TA Instruments, Greifensee, Switzerland). During the measurements, a small amount (~5 mg) of a sample was placed in an aluminum pan and heated from 25 to 600 °C. The TGA measurement was repeated twice for each composite. Dynamic mechanical analysis (DMA) was carried out using a DMA/SDTA861e analyzer (Mettler Toledo, New Castle, PA, USA). All the measurements were performed using the tension mode at 5 Hz from −80 to 80 °C, with a heating rate of 2 °C/min and a strain amplitude of 10 µm. For each composite, two specimens with the dimensions of 105 mm × 4 mm × 1 mm were used during DMA analysis. The standard deviation values of deteremined log E’ and tan δ were ±0.03 and ±0.02, respectively. FT-IR measurements were conducted using a Thermo Scientific Nicolet 6700 FTIR spectrometer (Waltham, MA, USA) to determine the carbonyl index (CI) values of the composites subjected to the aging process. The small pieces of the EN composite sheets were employed for this measurement. All the spectra were recorded with a 32-scan signal in the wavelength range of 600–4000 cm^−1^. The carbonyl index (CI) was calculated according to Equation (1) [22]:

CI = A_C=O_/A_–CH2–_, (1)

where A_C=O_ is the area of the carbonyl absorption band in the range of 1600–1800 cm^−1^ and A_–CH2–_ is the area of the internal reference band in the range of 3000–2800 cm^−1^. The mechanical properties of the EN composites were characterized according to the ISO-37 standard using a Zwick 1435 tensile testing machine (Zwick Roell Group, Germany). The tensile strength (TS) test was carried out for five standard dumbbell-shape samples (W-3 type), and the average values of TS have been applied for determination of aging factor. The aging factor values were obtained based on the following Equation (2) [23]:

Aging factor = (T_SA_·E_BA_)/(T_SB_·E_BB_), (2)

where T_SB_ and E_BB_ are the tensile strength [MPa] and elongation at breakage point [%] of the composites, and T_SA_ and E_BA_ are the tensile strength and elongation at breakage point after the aging process.

## 3. Results

### 3.1. Secondary Ion Mass Spectrometry (TOF-SIMS) and ^27^Al NMR Spectroscopy

**Figure 2:**
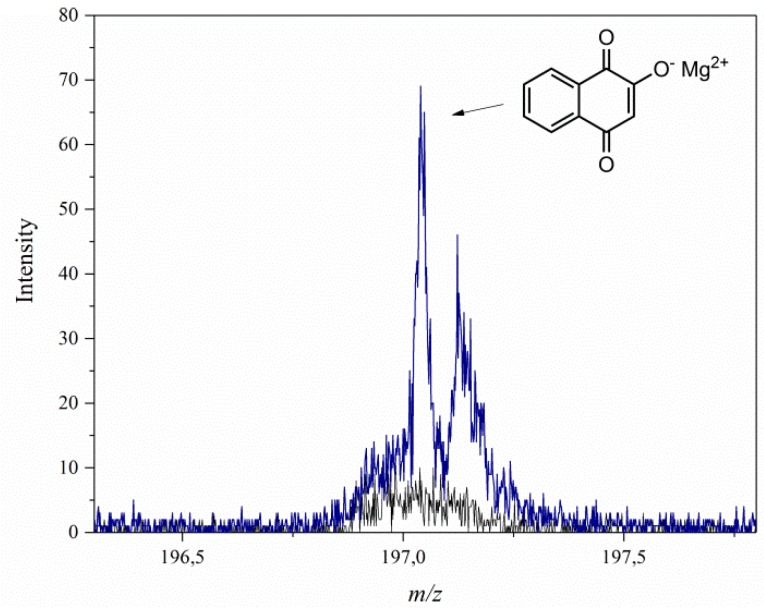
TOF-SIMS spectra of the LH (black line) and LH/lawsone pigment (blue line).

**Figure 3:**
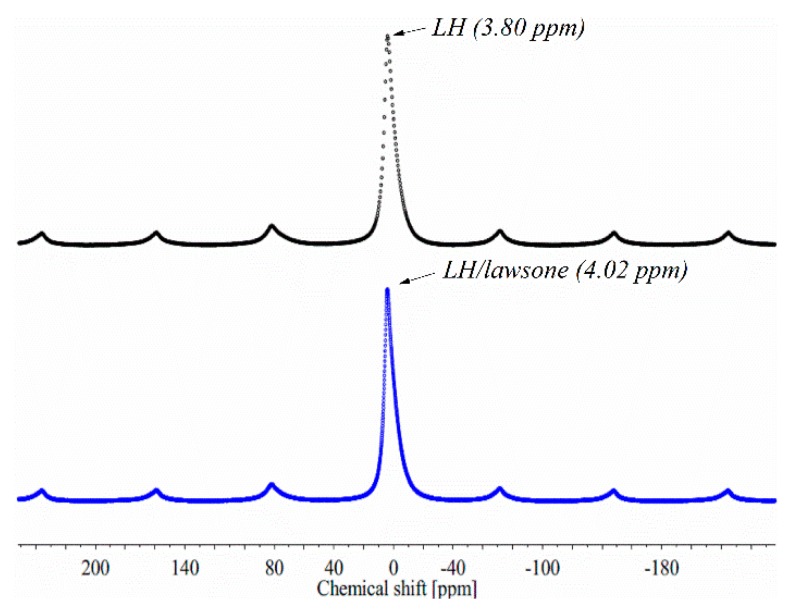
^27^Al MAS NMR spectra of the LH and LH/lawsone pigments.

The TOF-SIMS method was used to study host-guest interactions between the LH structure and the 2-hydroxy-1, 4-naphthoquinone. In our previous research, TOF-SIMS had also been used to investigate the stabilization mechanism of azo dye and anthraquinone chromophore (alizarin) by aluminum-magnesium hydroxycarbonate with different Al/Mg ratios [20,21]. Based on our previous results, it was expected that the lawsone molecules would be stabilized by ions (Mg^2+^ and/or Al^3+^) in the LH matrix, and that this would be visible on the spectra as characteristic ions emitted from the LH/lawsone complexes. The TOF-SIMS spectrum of LH/lawsone (Figure 2) indeed revealed the presence of a C_10_H_5_O_3_Mg^+^ ion peak at *m*/*z* 197. However, the TOF-SIMS spectra of the LH/lawsone pigment did not contain peaks corresponding to a complex of the dye with Al^3+^ ions. In fact, some Al^3+^ interactions were observed by NMR studies. The ^27^Al MAS NMR line positions tend to occupy the −5 to 15 ppm range for AlO_6_ sites [24]. Figure 3 shows the spectrum of the LH host and LH/lawsone pigment. Before modification, the LH sample showed a resonance peak at a chemical shift, δ, of ~3.8 ppm, whereas LH/lawsone showed a chemical shift in the resonance peak of 4.0 ppm. This was similar to the chemical shift obtained for alizarin stabilized by an LH host with lower magnesium content (3.4 to 3.2 ppm) [21]. As in our previous studies, modification of the LH host with lawsone dye was again observed to be accompanied by a decrease in the emission of CO_2_^−^ ions, confirming the presence of carbonate ions (CO_3_^2−^) in the LH structure. This was most likely a result of the complexation of C_10_H_5_O_4_^−^ ions with ions present in the LH host. These results correlate with further data obtained from thermogravimetric analysis (TGA) and suggest that the mechanism of hybrid pigment formation was similar to that for lake pigments obtained on traditional inorganic carriers, involving interactions between the dye and relatively insoluble alkaline earth salts [21,25].

### 3.2. Powder X-ray Diffraction (PXRD) and Scanning Electron Microscopy (SEM)

**Figure 4:**
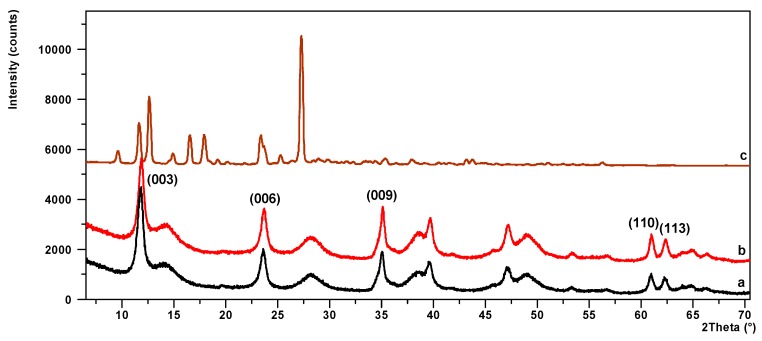
Powder XRD patterns for LH (**a**), LH/lawsone (**b**) and lawsone (**c**).

Figure 4 shows a sequence of X-ray powder diffraction patterns for LH, LH/lawsone and lawsone, respectively. The diffraction pattern of pure LH in Figure 4a consists of three sharp peaks at low 2*θ* angle, equivalent to diffraction by planes (003), (006) and (009). This indicates good crystallinity. The interlayer distances of (d003) and (d006), corresponding to 0.758 nm and 0.381 nm, are due to basal reflections indexed to a hexagonal crystal lattice with rhombohedral 3R symmetry. These values, together with other non-basal spacings at 0.152 nm (d110) and 0.149 nm (d113), are consistent with the typical XRD pattern of hydrotalcite-like compounds. On the slopes of the sharp peaks, small broad peaks can be observed which indicate the presence of co-precipitated, poorly crystalline boehmite. After the addition of 15% lawsone, the basal reflection (003) of the pigment (Figure 4b) does not change its 2*θ* angle. Unlike the azo dye, the LH/lawsone pigment was not observed on the XRD pattern as an additional crystal phase [20].

**Figure 5:**
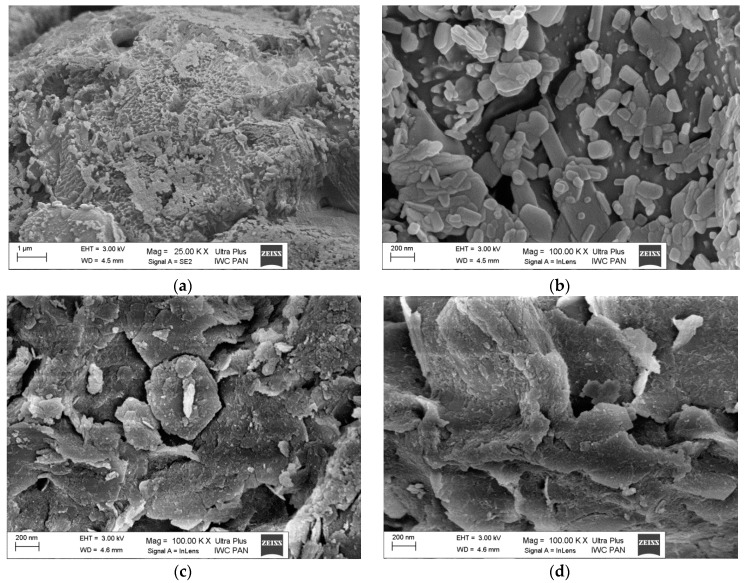
SEM micrographs of lawson (**a**,**b**), LH (**c**) and LH/lawson pigment (**d**).

Scanning electron microscopy (SEM) was used to study the morphology and grain size of the powders (Figure 5a–d). The unmodified LH host (Figure 5c) was characterized by an irregular layered structure and thin platelets with lateral dimensions of 400–700 nm up to a few μm. The SEM pictures of the lawsone chromophore confirmed the results of XRD and showed the presence of crystalline dye structures with irregular shapes ranging in size from 200 nm up to a few μm (Figure 5a,b). In contrast to the azo dyes and anthraquinone chromophore studied previously, lawson cannot clearly be seen on the LH surface after the stabilization process (Figure 5d) [20,26]. This may be explained by the fact that lawsone forms a relatively uniform layer on the LH surface.

### 3.3. Thermogravimetric Analysis (TGA)

**Figure 6:**
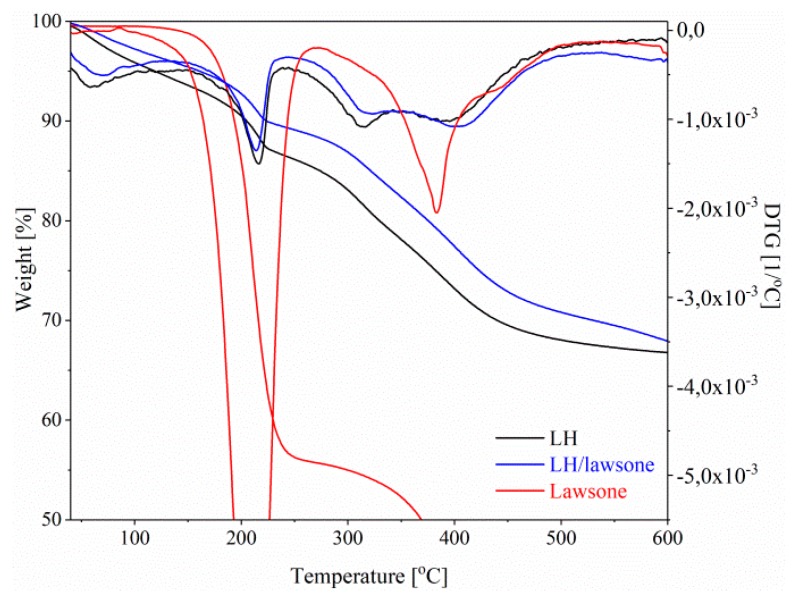
TGA/DTG curves for LH, LH/lawsone and lawsone.

Thermogravimetric analysis (TGA) was used to determine the thermal stability of the prepared hybrid pigment, LH host and unmodified lawsone sample. The TGA/DTG curves are shown in Figure 6. As can be seen, the TGA/DTG curves for the LH and LH/lawsone samples exhibit two main weight losses with four distinct steps. The first mass loss, observed below 100 °C, corresponds to the departure of physisorbed water molecules from the LH surface. The rapid weight loss at around 200 °C can be attributed to the removal of water molecules from the interlayer gallery of the LH. The next two mass loss steps between 300 and 450 °C are related to the dehydratation of hydroxyl groups from the LH layers, to loss of carbonate ions from the interlayer galleries and to the complete decomposition of metal hydroxide layers [27]. For pure lawsone, two weight losses may be observed and the main the thermal decomposition peak occurs at 210 °C. However, after the modification process the thermal stability of the pigment was enhanced significantly. The T_05%_ and T_10%_ of the LH sample increased from 120 °C and 206 °C to 161 °C and 224 °C (LH/lawsone), respectively. This confirms that the complexation of dye molecules with aluminum-magnesium hydroxycarbonate host results in a hybrid pigment with improved thermal stability. The reason may be the strong dye-metal interactions between the lawsone molecules and the LH host. Similar observations were made in our previous studies, most likely related to a decrease in the concentration of carbonate ions (CO_3_^2−^) and due to dye-metal complexation [20,26].

**Figure 7:**
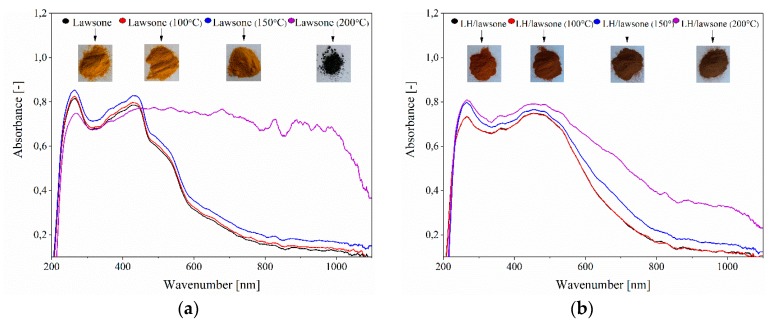
Color changes in lawsone (**a**) and lawsone pigment (**b**) after thermal aging at different temperatures.

The color stability of the LH/lawsone powder under elevated temperature was studied in an oven at 150, 200 or 250 °C for 30 min. Dye chromophore degradation is reflected in color variation, so UV–Vis spectroscopy was used to examine the decomposition of the samples. At 150 °C, the shape of the pure lawsone spectra was changed and at 200 °C carbonization of the dye was observed (black color), indicating degradation of the chromophore (Figure 7). This correlates with the results of TGA/DTG, as the main decomposition peak of 2-hydroxy-1,4-naphthoquinone started bellow 200 °C. The color stability of the LH/lawsone pigment was found to be enhanced, as much lower changes in color were detected at the same temperatures. Stabilization of the dye onto LH contributed to improve the thermal resistance of the tested pigment.

### 3.4. Mechanical Properties and Carbonyl Index of EN Composites

**Figure 8:**
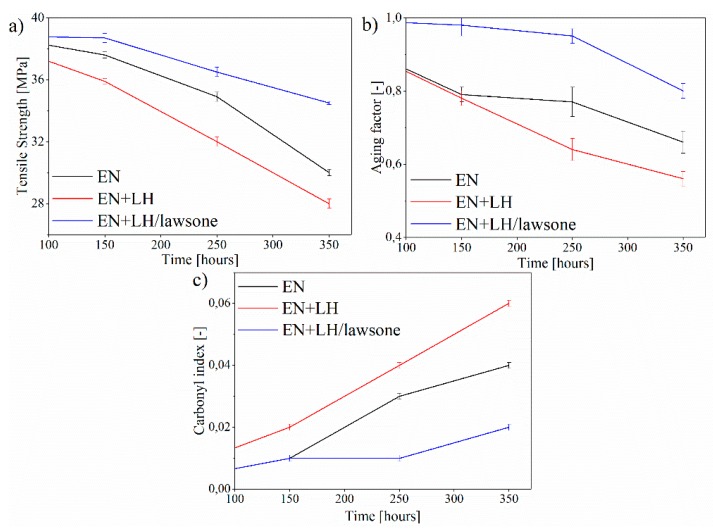
Tensile strength (**a**), aging factor (**b**) and carbonyl index (**c**) as a function of the aging time of EN, EN+LH and EN+LH/lawsone composites.

The mechanical properties of polymer composites are very sensitive to weathering. Therefore, a tensile test is a useful way to monitor the behavior of the samples under unfavorable aging conditions. Chemical changes produced by irradiation can cause the surface of EN materials to become brittle, with lower values for mechanical strength (TS) [28]. Before solar irradiation, the neat (EN and EN+LH) and EN+LH/lawsone materials exhibited TS in the range of 37–40 MPa. For EN and EN+LH materials, marked degradation occurred after 350 h of UV irradiation, leading to significant decreases in TS of 8 MPa and 10 MPa, respectively. The TS of EN+LH/lawsone was only slightly affected by 350 h of aging (36 MPa) (Figure 8a and Table S1 from Supplementary Material). The progress of degradation was also investigated as an ageing factor, that is, the dependence of tensile strength and elasticity before and after aging. After 350 h of irradiation, the aging factors for the reference samples (EN and EN+LH) were significantly lower than 1, which confirmed that their mechanical properties had undergone marked deterioration. In contrast to the EN and EN+LH films, the EN+LH/lawsone composite was only slightly affected by solar irradiation, as its aging coefficient was 0.8. The aging resistance of the EN composite was considerably improved by the LH/lawsone pigment. In a previous study, we had found that EN films containing LH hosts modified with azo chromophores were more resistant to UV photodegradation than composites filled with unmodified LH [29]. We attributed this finding to the UV shielding effect of hybrid pigments containing azo dye. Kutlu et al. [10] reported a similar effect in their work on dye-intercalated layered double hydroxide in polypropylene films. They suggest that dye may additionally interact with polypropylene, in such a way that does not involve UV absorbance, and which imparts additional durability to dye/PP nanocomposites. We conclude that the one of the main reasons for the enhanced durability of the EN+HP film was the UV absorption capability of the LH/lawsone in the EN copolymer.

Fourier transform infrared (FT-IR) spectroscopy was used to verify the chemical changes on the surface of the EN, EN+LH and EN+LH/lawsone composites after solar irradiation. The oxidation reactions promoted by aging led to the formation of carbonyl groups between 1790 and 1700 cm^−1^. These can be related to the formation of acid groups, ketones or esters [30]. Figure 8c presents the carbonyl index, calculated as the ratio of the carbonyl band intensity at 1710 cm^−1^ and the band intensity at 2918 cm^−1^. The carbonyl index is related to CH_2_ asymmetric stretching, and should not be strongly affected by degradation processes [31]. The highest values after 350 h were obtained for the EN and EN+LH films, indicating pronounced oxidation of the surfaces of those samples. The concentration of photooxidized products of the EN+LH/lawsone composite, also expressed in terms of the carbonyl index, was found to be considerably lower (~0.02). Thus, it is clear that the presence of the hybrid pigment in the EN copolymer led to higher stability against solar degradation.

**Figure 9:**
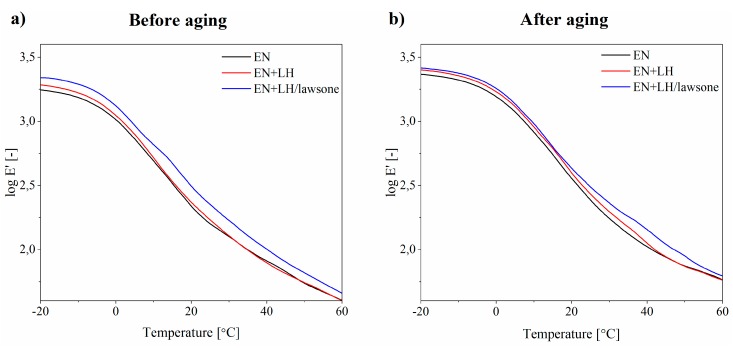
Temperature dependence of the storage modulus (E’) logarithm at 5 Hz for EN, EN+LH and EN+LH/lawsone composites before (**a**) and after (**b**) 350 h of aging.

**Figure 10:**
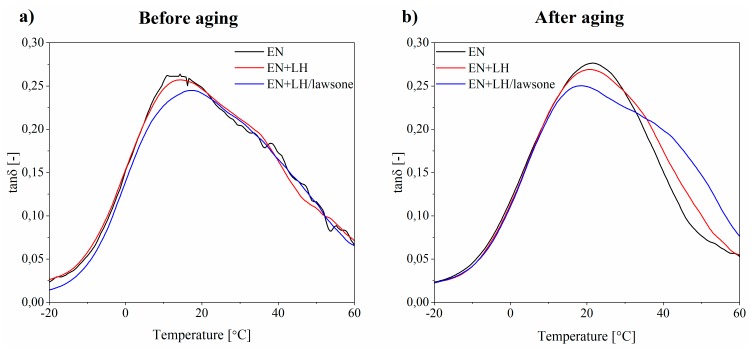
Temperature dependence of tanδ at 5 Hz for EN, EN+LH and EN+LH/lawsone composites before (**a**) and after (**b**) 350 h of aging.

The viscoelastic properties of EN composites exposed to the aging process were determined based on dynamic experiments over a range of temperatures. The results for storage (E’) modulus and loss factor (tan δ) are presented in Figure 9 and Figure 10.

When the loss factor (tan δ) is plotted against temperature, the glass transition temperature (*Tg*) is indicated by a peak. As shown in Figure 10, the *Tg* value of the EN+LH/lawsone (18 °C) sample was higher than that for both the neat EN copolymer (16 °C) and the LH-filled composite (17 °C). Moreover, the addition of 3 g of the hybrid pigment (LH/lawsone) reduced the tan δ peak height. This can be explained by the restricted movement of the polymer chains, due to good polymer-filler interactions. The storage modulus (Figure 9) of the composites was also found to increase upon addition of the hybrid pigment.

Increases in the storage modulus (E’) of the EN and EN+LH composites were observed following aging. For example, the storage modulus at 20 °C increased from 2.22 × 10^2^ MPa to 3.57 × 10^2^ MPa for the neat EN copolymer and from 2.33 × 10^2^ MPa to 3.89 × 10^2^ MPa for EN composite filled with unmodified LH. This effect can be attributed to the greater stiffness of these samples, as a result of the formation of photo-products and a crosslinked network during aging (it was also reflected in the decreased elongation at break values of these samples—Table S1, Supplementary Material). As a consequence, the composites became more brittle and lost flexibility. It is also well known that during the solar irradiation of polymer, the formation of three-dimensional networks by means of crosslinks leads to a decrease in chain mobility and an increase in the loss factor maximum [32]. This effect was not obvious in our study. However, the progressive aging of the reference and LH-filled EN compounds was reflected in the glass transition temperatures (*Tg*), which were shifted to higher temperatures due to changes in the polymer structure. Changes in the storage modulus of the EN+LH/lawsone composite after 350 h of irradiation were considerably lower (from 3.33 × 10^2^ MPa to 3.89 × 10^2^ MPa) than in the case of the reference composites at the same temperatures. This is consistent with the results of the tensile tests.

### 3.5. Thermal Stability of EN Composites

**Figure 11:**
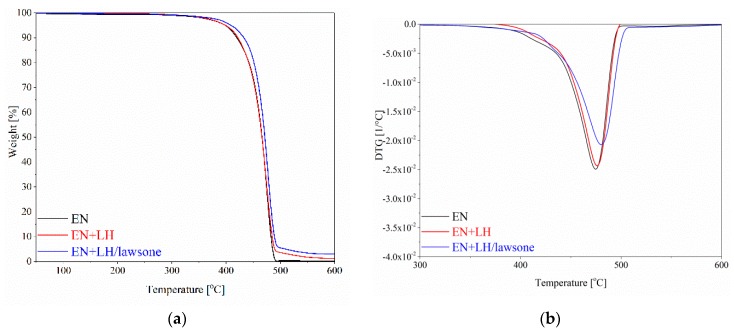
The TGA (**a**) and DTG (**b**) curves for EN, EN+LH and EN+LH/lawsone composites.

Thermal stability, determined conventionally by thermogravimetric analysis (TGA), is a very important factor in the characterization of polymer composite materials. Figure 11 shows the TGA/DTG thermograms of the pristine EN copolymer and its composites filled with 3 g of unmodified LH and hybrid pigment. The presence of the LH-based fillers caused distinct changes in thermal decomposition behavior in comparison to the unfilled EN. From the thermogravimetric data, it can be assumed that the thermal degradation of the reference samples began at lower temperatures (398 °C for EN and 399 °C for EN+LH) than in the case of the hybrid pigment (409 °C). Moreover, the decomposition temperatures at which 50% weight loss took place (T_50%_) for the filled composites were delayed compared to the reference. This may be related to the fact that LH layers are able to impede the internal diffusion of heat and gaseous products which form during the thermal degradation process. As a result, composites containing LH-based fillers may exhibit increased thermal stability compared to pristine polymer. Improvements in the thermal stability of EN-filled composites were also seen in the char residues from the samples. By incorporating 3 g of LH and LH/lawsone, the char yields of the samples were increased, due to the more thermally stable inorganic network.

## 4. Conclusions

In this study, we produced colored ethylene-norbornene films with improved aging resistance by the addition of organic-inorganic pigment. The hybrid pigment was synthesized by the incorporation of lawsone dye into an aluminum-magnesium hydroxycarbonate inorganic carrier. ^27^Al solid-state NMR and TOF-SIMS were used to confirm the interaction of 2-hydroxy-1,4-naphthoquinone with particular ions present in the LH matrix. TOF-SIMS demonstrated that complexation of the LH with lawsone had been successful and showed the presence of characteristic C_14_H_7_O_4_Mg^+^ ions. Interactions between Al^3+^ ions and dye chromophore were detected by ^27^Al NMR, with a chemical shift that was observed in the case of the hybrid pigment in comparison to the pure LH host. Color stability at elevated temperatures was enhanced compared to the pure dye. This was ascribed to metal-dye interactions and the effective transformation of lawsone into an organic-inorganic pigment. The slight decrease in mechanical strength and the low concentration of carbonyl groups in the EN+LH/lawsone composite indicate that the polymer with hybrid pigment was significantly less affected by solar irradiation than the reference samples (EN and EN+LH). The results of this study show that the hybrid pigment can play a dual role in EN composites, providing both an interesting color and marked protection against solar aging.

## Supplementary Materials

The following are available online at https://www.mdpi.com/2073-4360/11/4/723/s1, Figure S1: Mixing curves of virgin EN compound and EN filled with LH and hybrid pigment, Table S1: Mechanical properties of studied composites before and after UV aging.
