# Supplementary material for: Improved Aging Stability of Ethylene-Norbornene Composites Filled with Lawsone-Based Hybrid Pigment
Source: Polymers (Basel). 2019 Apr 19;11(4):723. doi: 10.3390/polym11040723 (PMC6523681; doi:10.3390/polym11040723)
Supplement: Supplementary file 1 [file polymers-11-00723-s001.pdf]

Supplementary material

Improved aging stability of ethylene-norbornene composites filled with lawsone-based hybrid pigment

Anna Marzec [1,]* and Bolesław Szadkowski [1]

[1] Institute of Polymer and Dye Technology, Faculty of Chemistry, Lodz University of Technology, Stefanowskiego 12/16, 90-924 Lodz, Poland
* Correspondence: anna.marzec@p.lodz.pl

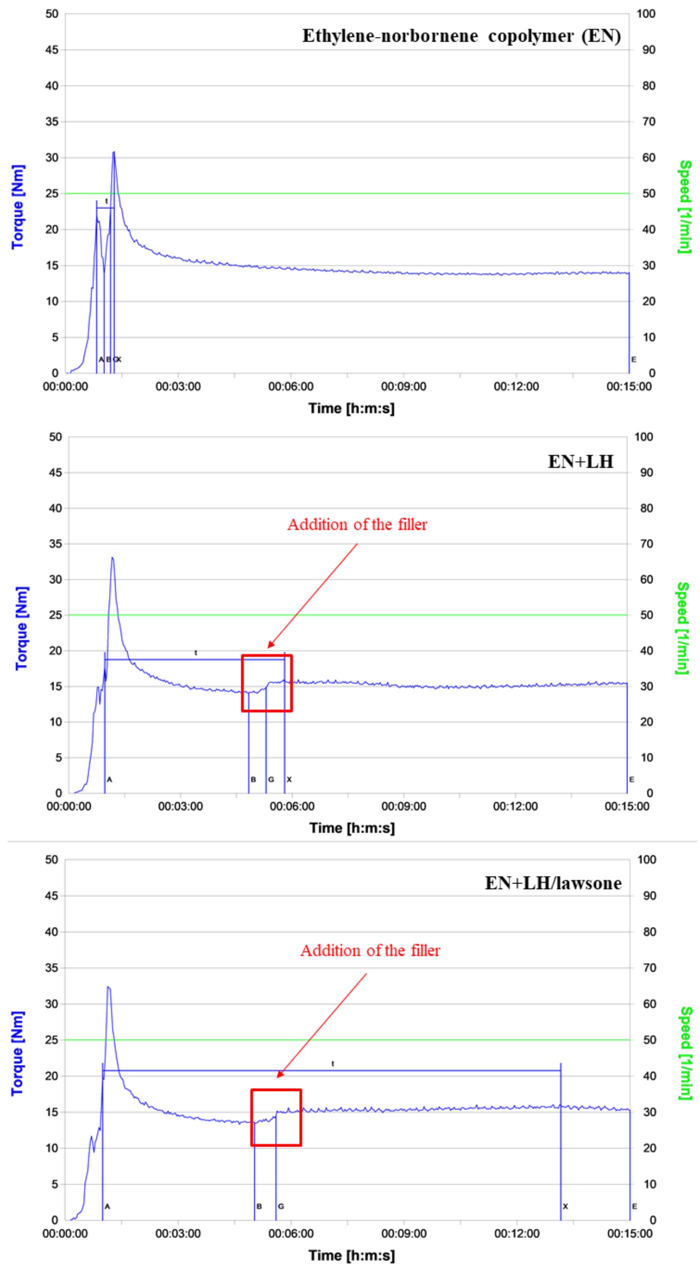

Figure S1. Mixing curves of virgin EN compound and EN filled with LH and hybrid pigment.

Table S1. Mechanical properties of studied composites before and after UV aging.

Polymer compound	T_{SB} [MPa]	E_{BB} [%]	150 h		250 h		350 h	
			T_{SA} [MPa]	E_{BA} [MPa]	T_{SA} [MPa]	E_{BA} [MPa]	T_{SA} [MPa]	E_{BA} [MPa]
EN	38.5	600	37.6	485	34.9	510	30.0	508
EN+LH	37.8	559	35.9	459	32.0	424	28.0	423
EN+LH/lawsone	38.9	551	38.7	543	36.5	558	36.0	476
